# Transcriptomic and functional analyses uncover a conserved effector driving genotype-dependent virulence in the *Sphaerulina musiva-Populus trichocarpa* interaction

**DOI:** 10.1128/mbio.03120-25

**Published:** 2026-04-16

**Authors:** Kelsey L. Søndreli, Tomás A. Rush, Milton T. Drott, Alistair McTaggart, William Alexander, Cole Sawyer, Hari B. Chhetri, Doug Hyatt, Robert Brueggeman, Jonathan K. Richards, Timothy L. Friesen, Daniel Jacobson, Paul E. Abraham, Joanna Tannous, Jared M. LeBoldus

**Affiliations:** 1 Department of Botany and Plant Pathology, Oregon State University 170397, Corvallis, Oregon, USA; 2 Biosciences Division, Oak Ridge National Laboratory 551173 , Oak Ridge, Tennessee, USA; 3 USDA-ARS Cereal Disease Laboratory 57840 , St. Paul, Minnesota, USA; 4 Centre for Horticultural Science, Queensland Alliance for Agriculture and Food Innovation, University of Queensland, Ecosciences Precinct 1974, Brisbane, Queensland, Australia; 5 Graduate School of Genome Science and Technology, University of Tennessee 312345, Knoxville, Tennessee, USA; 6 Crop and Soil Sciences, Washington State University 6760, Pullman, Washington, USA; 7 Department of Plant Pathology and Crop Physiology, Louisiana State University Agricultural Center 124524, Baton Rouge, Louisiana, USA; 8 Edward T. Schafer Agricultural Research Center, Cereal Crops Research Unit, US Department of Agriculture–Agricultural Research Service 700124, Fargo, North Dakota, USA; Cornell University, Ithaca, New York, USA

**Keywords:** virulence factors, effectors, *Septoria* canker, poplar, bioenergy

## Abstract

The introduction of invasive microbes compromises the structure, biodiversity, and function of naïve ecosystems. *Sphaerulina musiva*, a hemibiotrophic pathogen that causes leaf spot and stem cankers in *Populus* species, exemplifies an invasive fungal pathogen spread by human activities. However, the genetic mechanisms of pathogenicity and virulence are poorly understood, impeding mitigation strategies. We utilized RNA sequencing to identify fungal effectors linked to stem canker formation, informing the development of future strategies for effective disease management. Our analysis revealed 70 genes differentially expressed at 2 weeks and 110 genes at 3 weeks between inoculated trees and controls. Notably, the gene with the highest expression at 2 weeks and the second highest at 3 weeks was homologous to *Extracellular protein 2* (*Ecp2*). Complementary genome-wide association studies linked sequence polymorphisms in this locus to phenotypic variation in disease severity. Infiltration of *S. musiva* Ecp2 into *Populus trichocarpa* leaves induced necrosis in susceptible genotypes. Gene disruption using a CRISPR-Cas9 RNP system resulted in a genotype-dependent reduction of stem canker and disease severity. Tracing the evolutionary history of this effector across the fungal kingdom, we uncovered clade-specific gene-family expansions and orthologs in new species. These findings raise questions about the function and adaptive significance of these gene families in fungal lifestyles. Our study provides the first tractable target for breeding resistant poplar genotypes, addressing the challenges of managing *S. musiva* and uncovering mechanisms that drive its virulence, and provides deeper insights into the evolutionary dynamics of a conserved small-secreted protein with a diversity of functions.

**IMPORTANCE:**

*Populus* species, key feedstocks in the bioeconomy, are severely impacted by leaf spot and stem canker caused by *Sphaerulina musiva*. This disease diminishes biomass, reduces wood quality, and increases tree mortality, jeopardizing industrial sustainability. Invasion of *S. musiva* into naïve ecosystems exacerbates these challenges by disrupting ecosystem processes. Breeding resistant poplar genotypes has been the primary strategy to combat this pathogen, but it has remained unclear which molecular drivers of infection breeders should target. Our study makes a significant advance by identifying a key necrotrophic effector that increases *S. musiva* virulence in specific *Populus* genotypes. We identify clade-specific gene-family expansions of this effector that raise questions about the function of closely related genes. Our research elucidates the ecology and evolution of a small-secreted protein across the fungal kingdom while offering insights that enable host-breeding efforts to reduce the economic impact of *S. musiva*.

## INTRODUCTION

Fungi employ a range of strategies to interact with host plants, leading to outcomes that vary from beneficial interactions to disease (1). Even among pathogens, there is a spectrum ranging from biotrophs that extract nutrients without killing the host to necrotrophs that induce necrosis to extract nutrients (2). Some pathogens, known as hemibiotrophs, initiate their life cycle in a biotrophic phase, evading the host’s immune responses before transitioning to a necrotrophic phase, where they kill host cells to access nutrients and reproduce (3). Understanding the molecular mechanisms driving these lifestyles is crucial to determine how pathogens invade new environments and disrupt established ecosystems (4).

*Sphaerulina musiva* (syn: *Septoria musiva, Mycosphaerella populorum*) is an invasive hemibiotrophic pathogen that causes severe leaf spot and stem canker diseases in North American *Populus* species. These trees play a dual role as keystone species of forest ecosystems and as bioenergy crops (5). Originally endemic to Eastern North America, *S. musiva’*s expansion into new regions has introduced significant ecological and economic challenges. Leaf spot infections lead to premature defoliation, reducing the trees’ photosynthetic capacity, while stem cankers compromise tree health by girdling or weakening stems, increasing susceptibility to wind breakage and tree mortality (5, 6). Biomass losses from *S. musiva* infections have been reported to reach as high as 63% of total yield (7). These losses are influenced by factors like the susceptibility of specific poplar genotypes and the environmental conditions in a given year or region. A recent survey in the United States where 10,000 hectares of a highly susceptible poplar clone were planted revealed that over 90% of trees were infected, and 74% exhibited severe stem damage (8). Together, these studies underscore the persistent and substantial impact of this pathogen on plantations and potential impact on natural ecosystems. Thus, there is a critical need to understand how *S. musiva* successfully infects and colonizes *Populus* trees.

The mechanisms by which *S. musiva* evades its host immune system remain largely unexplored, representing a significant gap in our understanding of its pathogenicity and virulence. Fungal pathogens typically escape or suppress host immune responses through a variety of mechanisms (9). A key tactic involves secreting effector proteins that bind to specific host targets, giving the pathogen control over host defenses. For example, the rice blast fungus, *Magnaporthe oryzae*, which produces an effector protein that interacts with the host plant’s E3 ubiquitin ligase, impairs immune signaling pathways facilitating fungal infection (10). In contrast, some effectors are recognized by host resistance genes, activating a host’s resistance response (11). Some effectors are specific to certain pathogen species, while others are broadly conserved across a wide range of taxa (12). It has been hypothesized that these conserved effectors may function as virulence factors for one host-pathogen interaction and an avirulence factor for another (13). To date, the *Puccinia coronata-Cochliobolus victoriae-*“Victoria type” oats interaction is a well-documented example of this phenomenon (13). Characterizing the effector repertoires of fungal pathogens is essential to understanding the molecular basis of virulence, and the frequency of this phenomenon in plant-pathogen interactions (14).

The availability of fungal transcriptomes and secretomes has streamlined effector protein identification (15), with proteomics being one of the most effective methods for pinpointing pathogen-secreted components involved in disease development. Alternatively, transcriptome analysis of infected tissues can be used to identify differentially expressed transcripts during the infection process (16–20). Further bioinformatic characterization and functional validation can be performed to elucidate the potential roles of the identified genes or proteins. Several factors have hindered this characterization in tree-pathogen interactions, including the lack of reference genomes and annotations (19–21), difficulty in culturing and genetically manipulating the causal pathogens (22), and barriers to transforming and propagating the host (23). However, these problems have been overcome in the *Populus trichocarpa-S. musiva* pathosystem, as both the host and the pathogen have annotated reference genomes (24, 25). In addition, *P. trichocarpa* can be clonally propagated and transformed, and *S. musiva* can be cultured under laboratory conditions and manipulated using various molecular and genetic approaches (21, 26). These advances provide an opportunity to examine the mechanisms underlying this host-pathogen interaction.

A previous study identified a set of genes that enabled *S. musiva* to colonize woody tissue, which were absent in the closely related species, *Sphaerulina populicola* (27). The authors suggested that these genes were crucial for *S. musiva*’s colonization of woody tissue, but they did not explicitly test this hypothesis due to the lack of genetic tools for manipulating *S. musiva* at that time. More recently, several secreted effector proteins were identified in *S. musiva* and functionally characterized to elucidate their contribution to virulence (28), but no gene knockouts were conducted to confirm the hypothesized functions. Our group has recently adapted the CRISPR-Cas9 genome editing tool that utilizes Cas9/sgRNA ribonucleoprotein complexes (RNPs) to genetically alter *S. musiva* (26). The primary objective of this study was to leverage this genome editing tool to validate the function of a virulence factor identified through transcriptomic analyses during the formation of necrotic cankers on *P. trichocarpa* stems inoculated with *S. musiva*. Additionally, we conducted a phylogenetic analysis to extend our current understanding of this effector’s evolution, function, and distribution across the fungal kingdom. This approach not only enhances our understanding of how the effector has evolved but also offers crucial insights into its ecological significance, informing future disease management strategies and mitigating the impact of high-risk pathogens in natural and managed ecosystems.

## RESULTS

### Transcriptome analysis identifies a virulence factor highly expressed during stem canker formation

**Fig 1:**
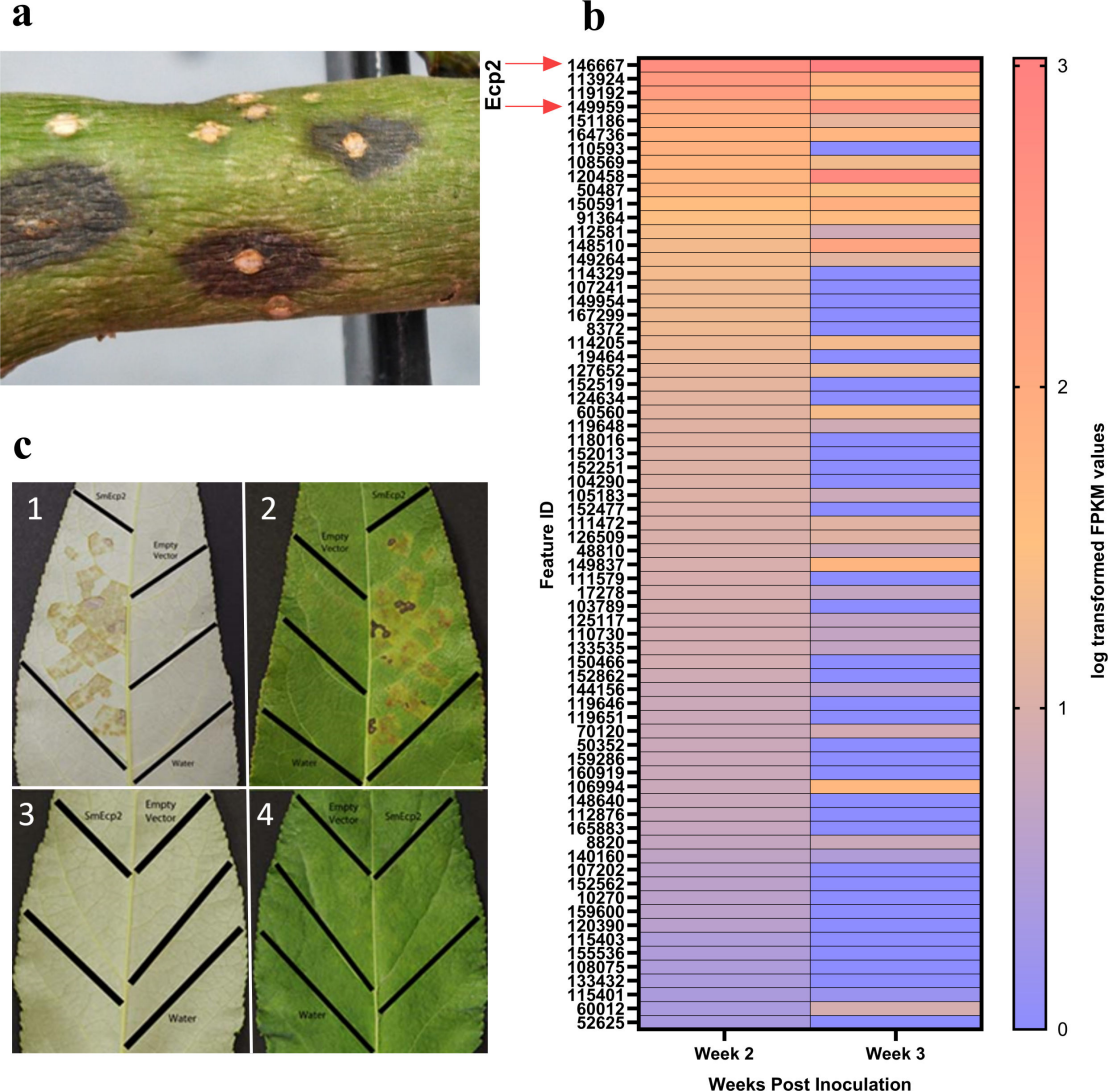
Identification of Ecp2 as an effector mediating virulence in *S. musiva.* (**a**) Cankers observed on *P. trichocarpa* stems inoculated with *S. musiva* at 3 weeks post-inoculation. (**b**) Heatmap of relative abundance levels of significantly upregulated *S. musiva* genes at 2 and 3 weeks post-inoculation. Expression levels are shown relative to *in vitro* controls cultured in KV-8 broth medium, highlighting genes that are overexpressed during infection at both time points. Darker shades of orange represent a higher level of abundance, while darker shades of purple signify lower expression levels. (**c**) *P. trichocarpa* genotype BESC 234 (1 and 2) infiltrated with *S. musiva* extracellular protein 2 (SmEcp2), empty vector control, and water control. HARC 26-5 (3 and 4) infiltrated with SmEcp2, empty vector control, and water control. Abaxial surface of the leaves (1 and 3) and adaxial surface of the leaves (2 and 4).

Six *P. trichocarpa* trees were point-inoculated with *S. musiva*. Water-soaked lesions first appeared around lenticels at 2 weeks post-inoculation (wpi) and became necrotic by 3 wpi (Fig. 1a). Lesions were collected at 2 wpi and 3 wpi, flash-frozen in liquid nitrogen, and mRNA was extracted. Three samples grown in liquid culture were included as a control. The total reads sequenced by the Ion Torrent PGM platform were 4,198,267, 4,915,453, and 3,125,004 for poplar stem cankers sampled at 2 and 3 wpi, and the fungus grown in liquid culture (control), respectively. Of the reads, 2.8% for 2 wpi and 7.2% for 3 wpi were mapped to the *S. musiva* reference genome (version 1). Similarly, 53.8% for 2 wpi and 50.2% for 3 wpi were aligned to the *P. trichocarpa* (version 3) reference genome (Table S1). Expression analysis of the fungal reads identified a total of 70 genes at 2 wpi and 110 genes at 3 wpi that were upregulated in the inoculated trees compared to the control to identify potential virulence factors present during canker formation (Fig. 1b; Data set S1). Most differentially expressed genes encoded hypothetical proteins with no known function (83% at 2 wpi; 80% at 3 wpi). Thirty-four of these genes were upregulated at both time points. Signal peptide cleavage sites were found in 69% (2 wpi) and 67% (3 wpi) of the sequences (Data set S1).

Five criteria were used to select a putative effector for cloning, protein expression, and infiltration: (i) a gene upregulated in comparison to the control; (ii) a gene with predicted functions or conserved domains indicative of effector activity; (iii) the presence of a predicted secretion signal cleavage site; (iv) the presence of the transcript at both 2 wpi and 3 wpi; and (v) the translated protein predicted to be <20 kDa in size. Using the five criteria outlined above, the gene with the highest transcript levels at 2 weeks post-inoculation (496.77 reads per kilobase per million [RPKM]) and the second highest at 3 weeks (1,055.71 RPKM) encoded for Extracellular protein *2* (Ecp2), a 19 kDa protein with a secretion signal, suggesting its involvement in extracellular processes. Consequently, *ecp2* was selected for further study, including protein expression through *Pichia pastoris* transformation and infiltration, to better understand its function and potential role in pathogenicity (Fig. 1b).

### Genome-wide association mapping validates Ecp2 as a candidate virulence factor

To complement the transcriptome analysis, we conducted genome-wide association studies (GWAS) analyses using phenotypic data for stem canker severity on two *P. trichocarpa* genotypes (GW9823 and CMBF28). SNP markers significantly associated with disease traits were identified and visualized in a Manhattan plot (Fig. S1). The most significant peak was located on chromosome 1 and corresponded to the same locus encoding Ecp2, which was also highlighted by the transcriptome analysis as one of the most highly expressed genes during stem canker development (Fig. 1b). A minor signal was detected at an *ecp2* paralog on chromosome 6, but it did not exceed the genome-wide significance threshold and was not considered biologically meaningful (Fig. S1).

### Infiltration assays validate the role of Ecp2 in disease development

Symptom development (necrosis) following the SmEcp2 infiltrations into *P. trichocarpa* leaves varied among genotypes (Table 1). In both experiments, necrosis developed 7–14 days after infiltration (Fig. 1c). Infiltrations with both water and empty vector controls did not produce symptoms (Fig. 1c). The infiltration results were consistent in both experiments (Table 1). Necrosis occurred only in susceptible genotypes; however, not all susceptible genotypes produced necrosis when infiltrated (Table 1).

**TABLE 1:** Infiltration results from both experiments showing each *Populus trichocarpa* genotype and the response from infiltrations of SmEcp2^*d*^

Genotype	Experiment 1	Experiment 2	Cankers per cm^*a*^	% Necrotic area^*b*^
BESC 184	−	−	0.845	14.722
BESC 22		−	0.037	4.663
BESC 234	+	+	0.700	11.227
BESC 801		−	0.514	10.446
GW 9805		+	0.402	8.233
GW 11024	+		0.583	15.307
HARA 26-2	−		0.000	N/A^*c*^
HARC 26-5	−	−	0.300	14.067
HOMA 21-5	−		0.634	6.487
SLMC 28-2	−		0.000	7.612

^*a*^ Cankers per cm were calculated from a previous experiment by counting the number of cankers on the stem and dividing it by the height (cm) at time of inoculation.

^*b*^ Percent necrotic area was calculated from a previous experiment by measuring the total necrotic area on each leaf and dividing by the total area of the leaf.

^*c*^ N/A = no data were available for that genotype.

^*d*^ Gray boxes indicate genotypes that were not used in each experiment. +, necrosis; −, no necrosis.

### Disruption of the *Ecp2* gene in *S. musiva* by CRISPR-Cas9-mediated homology-directed repair (HDR)

**Fig 2:**
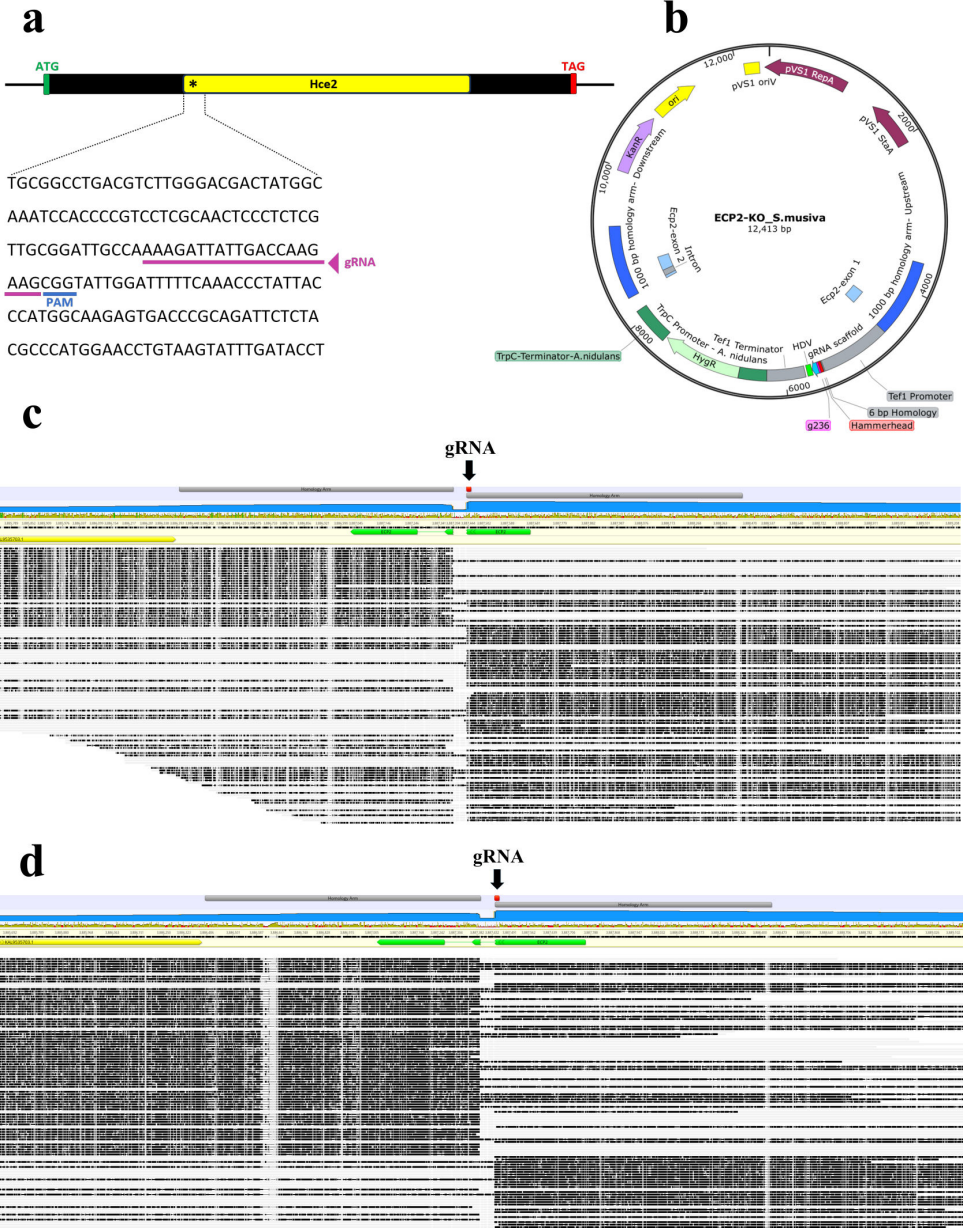
Disruption and validation of the *ecp2* gene in *S. musiva* strain MN-14. (**a**) Schematic representation of the gRNA design on the Hce2 conserved domain of the *ecp2* open reading frame. (**b**) Map of plasmid ECP2_KO_S.musiva. This plasmid consists of the *ecp2* deletion cassette, constituted of a gene conferring resistance to hygromycin under TrpC promoter and terminator from *Aspergillus nidulans,* flanked by 1,000 bp homology arms to allow homology-directed repair at the gRNA cut site. Sequencing of the genomes of the Δ*ecp2* strains 1 (**c**) and 5 (**d**) reveals disruption of the *ecp2* locus by CRISPR-Cas9-mediated knockout at the gRNA cut site. A tandem insertion of the *hph* cassette was observed in Δ*ecp2*-10, which prevented reliable alignment and is therefore not shown.

Protoplasts of the *S. musiva* strain MN-14 were co-transformed with the gRNA/Cas9 nuclease complex (Fig. 2a) and donor DNA (Fig. 2b). Twenty transformants were randomly selected on SMM plates supplemented with 100 µg/mL hygromycin. The negative control experiments did not result in hygromycin-resistant colonies. Eighteen out of 20 transformants were chosen for their ability to grow on a second round of selection plates. To confirm that a mutation occurred at the target site, two fragments at the 5′ and 3′ ends of the *ecp2* gene were amplified by PCR using primer sets ECP2-KO_conf5′F/R and ECP2-KO_conf3′F/R, respectively. For these PCRs, one of the primers was designed to anneal outside the deletion cassette, and the other within the selectable marker. The expected amplicons were obtained in 15 tested transformants (data not shown). An additional PCR targeting the *ecp2* open reading frame (ORF) was performed using the primer set ECP2-KO_ORF_F/R. Each monoconidial line yielded a single PCR product of 4,096 bp, corresponding to the disrupted *ecp2* ORF. In contrast, the wild-type (WT) control strain produced a 407 bp amplicon, indicative of the intact ORF (data not shown). The exclusive presence of the larger amplicon in the monoconidial lines suggests successful gene disruption and supports the conclusion that these lines are homokaryotic. We further validated the disruption of the *ecp2* gene and the absence of Cas9 off-target effects in three randomly selected transformants (Δ*ecp2*-1, 5, and 10) through nanopore whole-genome resequencing . Long reads generated from the minion for both isolates MN14 Δ*ecp2*-1 and Δ*ecp2*-5 were initially mapped using minimap2 (version 2.24) with default parameters to the donor DNA. This subset of reads, 149 reads for Δ*ecp2*-1 and 409 reads for Δ*ecp2*-5, was then used to map against the reference genome for WT strain MN14 (GCA_052057135.1) using minimap2 and output into sam format via samtools (version 1.17). Indexed and sorted reads were then imported into Geneious (version 2025.0.3) for visualization using the MN14 genome as reference. Sites with over 50% disagreement to the reference genome were hidden for clarity using Geneious. The majority of reads for both isolates map to chromosome 1, between coordinates 3,879,000 and 3,982,000, which corresponds to the *ecp2* reading frame (green) and surrounding genes (yellow) (Fig. 2c and d). Further inspection of the alignment denotes a drop in read coverage (blue plot) immediately following the known gRNA cut site (red) spanning coordinates 3,887,395 to 3,887,439. This location corresponds to our chosen insertion site, and the donor DNA homology arms have been highlighted for reference (gray). Reads for both isolates contain high levels of clipping (grayed-out reads) where the alignment to the reference genome (black read) no longer matches, which correspond to the donor DNA. Given the high density of reads at the ecp2 reading frame, which originally map to the hygromycin cassette, the level of clipping associated with DNA misalignment as would be the case with a large insertion, and the low number of reads which map to other chromosomes in the genome, we conclude that the donor DNA inserted a single time in the genomes of both isolates, representing clean knockouts of the *ecp2* gene. In Δ*ecp2*-10, a tandem insertion of the hygromycin deletion cassette was observed at the *ecp2* locus. Statistical metrics derived from whole-genome sequencing of the three mutant strains are summarized in Table S2.

Despite many attempts, we were unsuccessful in obtaining a correct complement of any Δ*ecp2* mutant with the *ecp2* gene. We thus evaluated three distinct Δ*ecp2* knockout strains for the following experiments to offer strong evidence that the phenotypic and biological effects observed were related to *ecp2* loss of function.

### Disruption of *ecp2* in *S. musiva* significantly alters the growth and sporulation *in vitro*

**Fig 3:**
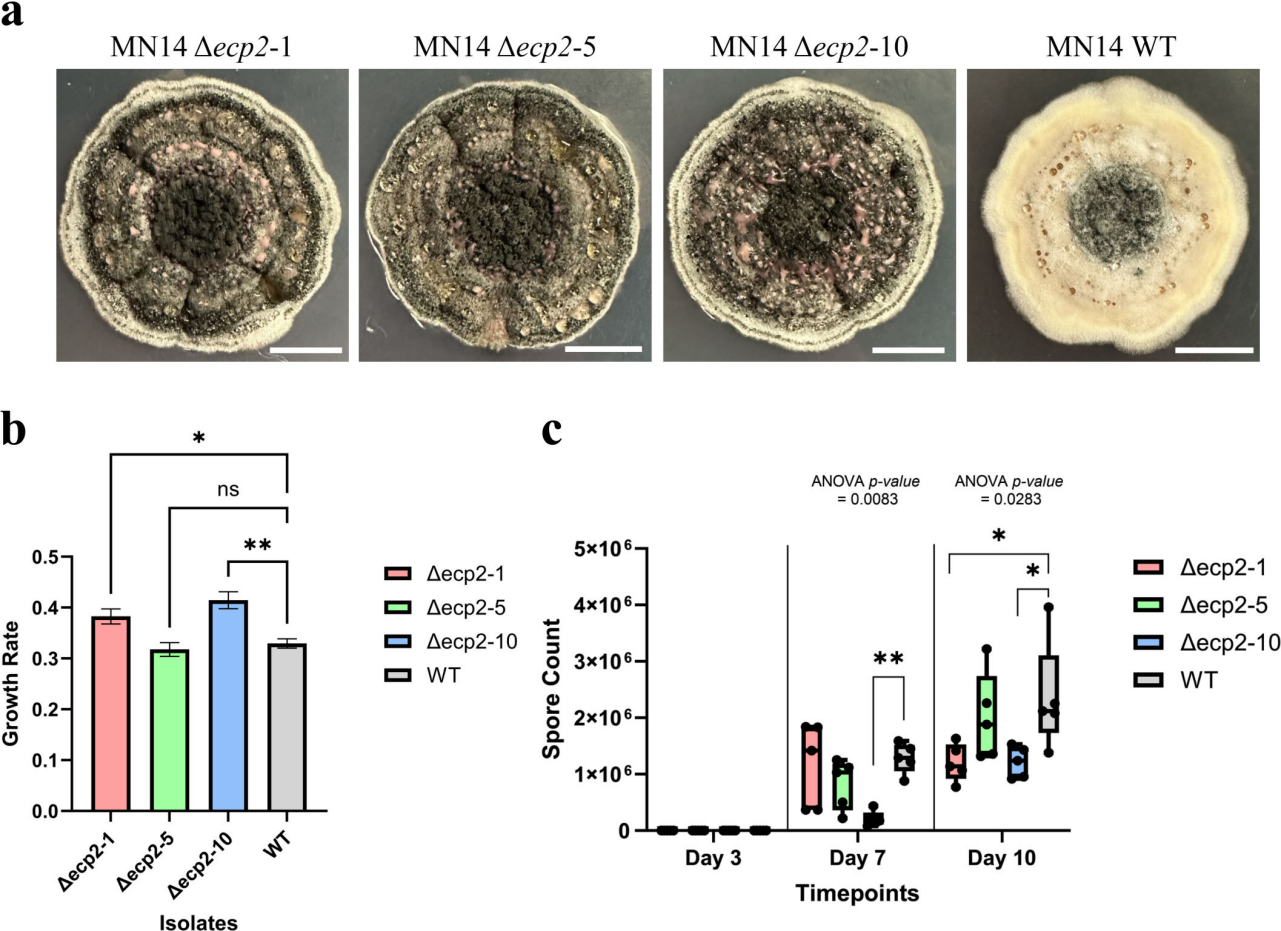
Disruption of *ecp2* affects both morphological and physiological characteristics of *S. musiva* strain MN-14. (**a**) Colony aspect of *S. musiva* WT and mutant strains grown on PDA for 10 days at 21°C. (**b**) Growth rates for each strain are over 10 days on PDA. (**c**) Spore counts for each strain at 3, 7, and 10 days post-inoculation on PDA. The number of spores was assessed per standard area sampled by plugging the fungal colony using the shaft-attaching end of a P1000 pipette tip. A one-way analysis of variance (ANOVA) was performed for both the growth and sporulation data. Dunnett’s multiple comparison test was used and compared to the WT strain. **** indicates a *P*-value <0.0001, *** indicates a *P*-value <0.001, ** indicates a *P*-value <0.01, * indicates a *P*-value <0.05, and no asterisk indicates not significant.

To assess the impact of the insertional mutagenesis of *ecp2* on *S. musiva’*s physiology, the three knockout mutants were first evaluated for disease-associated phenotypes such as sporulation and mycelial growth *in vitro*. Overall, the *ecp2* disruption resulted in a noticeable variation in colony appearance when inoculated at the center of PDA plates (Fig. 3a). Phenotypic changes were consistent across all three distinct Δ*ecp2* mutants.

Interestingly, the disruption of *ecp2* resulted in a significant increase in vegetative growth rate in two out of three mutants (Δ*ecp2*_1 and Δ*ecp2*_10; Fig. 3b) while also causing a substantial reduction in spore production at both 7 and 10 days post-inoculation in these strains. Although Δ*ecp2*_5 also showed a reduction in sporulation compared to the WT at the same time points, the difference was not statistically significant, and its growth rate remained unaffected (Fig. 3b and c).

### Disruption of *ecp2* in *S. musiva* results in *Populus* genotype-dependent reduction of stem canker formation and disease severity on leaves

**Fig 4:**
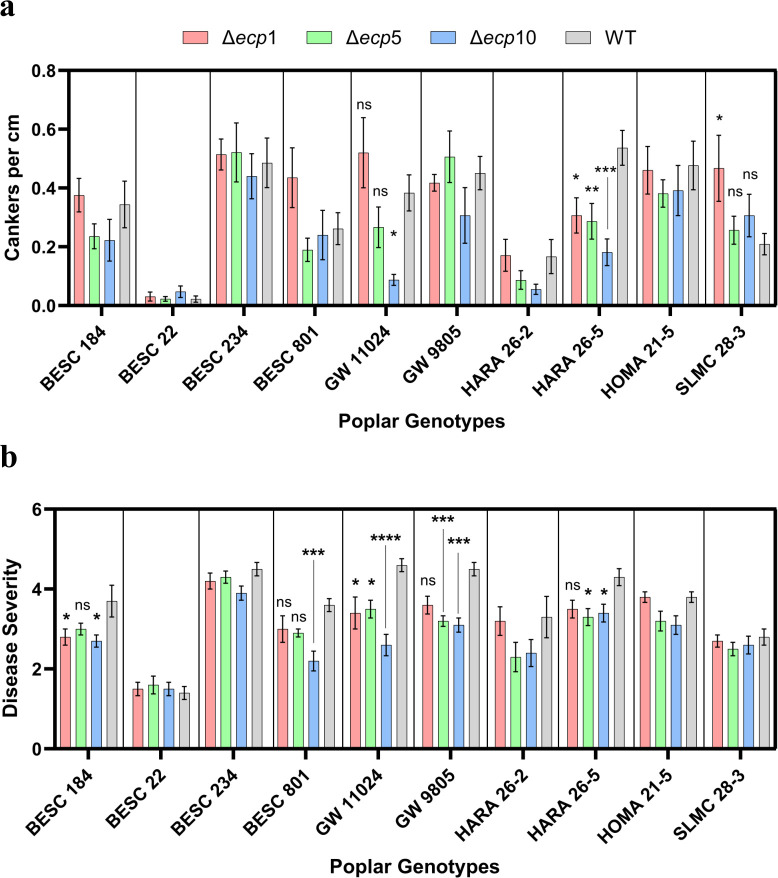
Disruption of *ecp2* in *S. musiva* strain MN-14 significantly reduces pathogenicity on certain *P. trichocarpa* genotypes. Canker size (**a**) and disease severity (**b**) recorded at 21 days post-inoculation with *S. musiva ecp2* mutants and WT strains. For the stem cankers data, genotypes BESC184, BESC22, BESC801, GW9805, and HARA 26-5 did not have a normal distribution, and the Kruskal-Wallis test was performed. For the other genotypes, a one-way ANOVA was used to compare the ability of each mutant and WT *S. musiva* strain to cause cankers on a single genotype. Genotype GW11024 had an ANOVA *P-*value = 0.0025. An outlier was detected through the ROUT method with Q = 1% and removed from the WT data set. Genotype HARA 26-5 had an ANOVA *P-*value = 0.0008. The other genotypes did not have a significant *P-*value. *P*-values are represented by asterisks as follows: <0.05 is *; <0.01 is **, <0.001 is ***, and <0.0001 is ****. NS indicates treatment was not significantly different from the control where *P-*value >0.05. Disease severity data is based on a 1-to-5 disease severity scale previously described in reference 29. Genotypes BESC184, BESC22, GW11024, GW9805, HARA 26-5, HARA 26-5, HOMA 21-5, and SLMC 28-3 did not have a normal distribution, and the Kruskal-Wallis test was performed. For the other genotypes, they had a normal distribution, and a one-way ANOVA test was performed. BESC 184, BESC 801, GW 11024, GW 9805, HARA 26-5, and HOMA 21-5 had approximate significant *P*-values of 0.0208, 0.0016, <0.0001, 0.0001, 0.0169, and 0.0265, respectively. *P*-values are represented by asterisks as follows: <0.05 is *; <0.01 is **, <0.001 is ***, and <0.0001 is ****. NS indicates treatment was not significantly different from the control where *P*-value >0.05. BESC 22, HARA 26-2, and SLMC 28-3 were not significant, and no *post hoc* analysis was conducted. The effect of treatments across poplar genotypes was not examined.

To investigate the role of ECP2 in mediating host susceptibility and disease development, we assessed the impact of *ecp2* disruption on stem canker formation and disease severity across 10 biological replicates of 10 distinct *P. trichocarpa* genotypes. Among the 10 genotypes tested, only HARA 26-5 and GW 11024 exhibited significant differences in canker development between plants inoculated with the Δ*ecp2* mutant strains and the WT strain (Fig. 4a). However, the pattern of significance varied between these two genotypes. In HARA 26-5, the disruption of *ecp2* resulted in a consistent and significant reduction of canker formation (cankers per cm) across all three independent Δ*ecp2* mutants (Fig. 4a). In contrast, in GW 11024, a significant reduction in canker formation was only observed in one of the mutants (Δ*ecp2*-10), while no significant differences were found between the other two mutants (Δ*ecp2-*1 and Δ*ecp2*-5) and the WT strain. No consistent effects were observed in the remaining genotypes, suggesting that the role of ECP2 in canker development may be genotype dependent. While our analyses indicate that the tandem insertion of the hygromycin deletion cassette in Δ*ecp2*-10 does not disrupt any annotated genes, we acknowledge that such insertions might have contributed to the significant reduction in canker formation observed in this transformant. In addition to the stem canker assessments, we also measured disease severity across the same genotypes. Notably, a reduction in disease severity was observed in the mutants compared to the WT in 5 out of 10 poplar genotypes, but the extent of this reduction was dependent on the specific Δ*ecp2* isolate. For example, in genotypes BESC 184, GW 9805, and HARA 26-5, two out of three Δ*ecp2* mutants exhibited a statistically significant reduction in disease severity, while the reduction in the third isolate was not statistically significant compared to the WT. In contrast, in genotype GW 11024, the reduction in disease severity was significant across all three Δ*ecp2* isolates (Fig. 4b).

### Paralogs of *ecp2* occur in Ascomycota with a gene-family expansion evident in Mycosphaerellaceae

**Fig 5:**
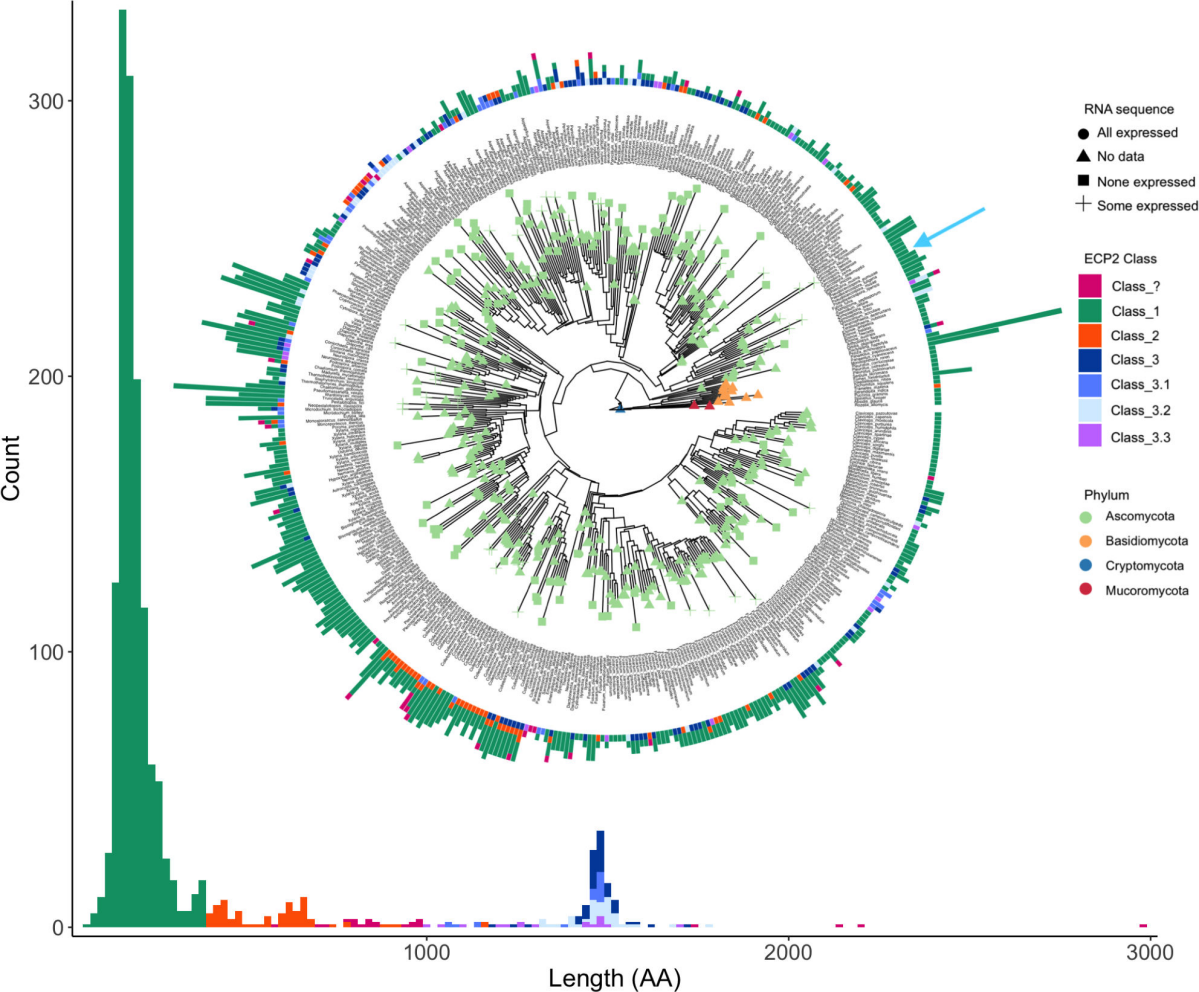
ECP2 orthologs are present across the fungal kingdom but are most common and diverse in Ascomycota. The phylogenetic relationship of all publicly available annotated fungal genomes that were found to contain at least one ECP2 ortholog is depicted in a quartet-based phylogeny, as modified from previous work (30). The full phylogeny, including genomes lacking ECP2 orthologs, is available in Fig. S2. The tree has been subsetted to contain only a single representative of each species based on the highest ECP2 ortholog count. Counts of different ECP2 class variants (see Materials and Methods) are depicted in a stacked bar graph around the outer rim of the phylogeny. The phylogeny is rooted at the only representative of the early-divergent lineage of Cryptomycota, *Rozella allomycis*, a species whose genome contains a single Class 1 ECP2 ortholog. In cases where RNA-seq was available for a species displayed in the tree, we compared the raw genomic counts of ECP2 orthologs between genomes and transcriptomes to determine if any, some, or all genes may be expressed. Analysis of RNA-seq data demonstrates that expression of ECP2 orthologs is widespread across the phylogeny. However, because of technical constraints, this analysis was performed at the species level and does not reflect a direct pairing of specific orthologs; we found no instances in which there were more ECP2 orthologs in expression data than in genomic sequences. The position of *S. musiva* in the tree is indicated with a blue arrow. A histogram of ECP2 orthologs is displayed as a function of protein length and corresponds to the same protein classes depicted on the phylogeny.

After characterizing the role of ECP2 in the *S. musiva-Populus* pathosystem, we extended our focus to examine the evolution of the *ecp2* gene family across the fungal kingdom. Specifically, we aimed to identify the distribution of ECP2-like effector classes and identify lineages where gene-family expansions had occurred, to infer what fungal ecologies may maintain or select for ECP2 diversity (e.g., if ECP2 orthologs have expanded outside of plant pathogenic lineages, this suggests novel ecologies that are not yet well understood). This analysis was conducted using a final data set comprising one representative genome from each of 1,389 species. ECP2 orthologs were mainly found in Ascomycete lineages (Fig. 5) and were most abundant in Sordariomycete genomes, with more than 96% of the 294 species having an average of 3.4 orthologs per genome. Eurotiomycetes and Dothideomycetes had ECP2 orthologs in roughly 50% of genomes, with slightly less than one copy per genome on average (185 and 151 genomes in the data set, respectively). Three of eight Orbiliomycetes had orthologs. The Ascomycete classes Leotiomycetes, Lecanoromycetes, and Pezizomycetes all had orthologs in less than 10% of genomes (*n* = 73, 50, and 23 genomes, respectively). While we did not find ECP2 orthologs in any other Ascomycete class, the distribution of these genes across this phylum seems to suggest a complicated history of gene-family expansion and contraction (Fig. S2). Outside of Ascomycota, ~8% of Mucoromycetes from Mucoromycota (24 genomes in the data set) and 5% Agaricales from Basidiomycota (*n* = 237) had ECP2 orthologs. The genomes of a single species from Pucciniomycetes in Basidiomycota (*Puccinia graminis*) and the only representative of Rozellomycetes in Cryptomycota (*Rozella allomycis*) each contained one ortholog. ECP2 orthologs were relatively rare outside of Ascomycota, with a patchy taxonomic distribution (Fig. S2).

ECP2 orthologs were found in high copy numbers in many genomes (Fig. 5). Interestingly, the Basidiomycete, *Panaeolus papilionaceus,* had the highest total number of these genes with 21 copies of the Class 1 variant. The Ascomycete, *Wardomyces moseri,* had the second highest copy number with 17 Class 1 variants. *Sordaria macrospora* had the third most copies with 12 Class 1, one Class 3, and one Class 3.1 orthologs. In general, Class 1 orthologs were the most common, widespread, and expanded class. Class 2 orthologs were found in only 74 genomes across Dothiodeomyecetes, Eurotiomycetes, and Sordariomyecetes. This class was most common in Sordariomycetes, where 52 genomes (29 of which were *Colletotrichum* species) were found to contain at least one Class 2 ortholog. While sampling bias in which genomes have been sequenced may obfuscate the true distribution of orthologs across the kingdom, we speculate that our large sample of genomes accurately reflects overarching patterns. In the Basidiomycota, we found a single Class 2 ortholog in the genome of the Agaricomycete, *Serendipita indica*. Class 3 orthologs were only found in Ascomycota where they were most common in Sordariomycetes and Eurotiomycetes (with some representation in Dothideomycetes, Leotiomycetes, and Orbiliomycetes). Our analysis of available transcriptomic data supported the idea that expression of ECP2 is widespread among diverse Ascomycete taxa with different complements of ECP2 orthologs. However, our RNA-seq data set had no representation outside of the Ascomycota, limiting our interpretation of these results.

Our classification of ECP2 orthologs is consistent with past work (31) and was successful in assigning a class to 97% of the 1,690 proteins in our data set. While there does appear to be a small peak in the histogram separating Classes 1 and 2 (Fig. 5), the length-based cutoff separating these classes is somewhat arbitrary; to the best of our knowledge, there is no known functional difference between these groups. A large portion of the 50 unclassified proteins (denoted “Class?”) were similar in length to Class 2 orthologs. These unclassified proteins often contain domains that have not previously been described in ECP2 orthologs. As most of these domains were present in only a single or a small number of proteins, we are hesitant to interpret the significance of these genes, as they may represent errors in annotation or PFAM identification. Class 3 variants (3.1, 3.2, and 3.3) included proteins that met the length requirements of Class 3 proteins but contained only a subset of associated protein domains. No proteins containing only the Class 3 defining domains were shorter than our 1,000-amino-acid cutoff.

## DISCUSSION

The invasion of fungal pathogens represents a serious threat to biodiversity and ecosystem function, especially for naïve host populations lacking evolved defenses against such threats (32). Therefore, understanding the molecular basis of host-pathogen interactions is critical to mitigate the impact of these invasive species. A key aspect of these interactions is the secretion of fungal effector proteins, which play pivotal roles in manipulating host physiology to facilitate infection (33). In necrotrophic pathogens, these effectors induce cell death and tissue breakdown, driving necrotic lesion and canker formation (2). As such, characterizing these effectors is key to understanding canker formation and disease development. Studying these mechanisms often involves cultivating pathogens in liquid media to secrete metabolites and effectors, which are then tested on host plants to assess responses. For example, culture filtrates from *Parastagonospora nodorum* and *Pyrenophora tritici-repentis* induced necrosis in wheat (34, 35). However, some effectors are produced only *in planta* (36), as demonstrated by *S. musiva* extracts being unable to induce necrosis in poplar (data not shown), suggesting that host presence is essential for activating effector-encoding genes. To test this hypothesis, we conducted RNA-seq to identify potential virulence factors involved in canker formation by comparing differential expression, at two separate time points, between symptomatic inoculated trees and the fungus grown in liquid culture.

An analysis of the expression pattern of genes, differentially expressed during infection, revealed a highly expressed gene with homology to a known necrotrophic effector Ecp2 was first isolated and characterized in the model *Cladosporium fulvum*-tomato interaction (37) where it was shown to contribute to virulence (38) and was capable of eliciting Cf-Ecp2-mediated HR when recognized by a tomato line carrying the corresponding Cf-Ecp2 resistance gene (39). Similarly, in the *S. musiva-Populus* interaction, SmEcp2 appears to function as a virulence factor facilitating disease development, although a cognate R-gene recognizing this effector has not yet been identified. More recently, Ecp2 orthologs carrying the necrosis-inducing factor domain (PF14856.7 in the Pfam database) have been identified in *Valsa mali*, the causal agent of apple cankers. Knockout studies have confirmed their association with pathogenicity on *Malus domestica* (apple) (40). In our study, the purified SmEcp2 protein induced necrosis in some genotypes of *P. trichocarpa* (Table 1; Fig. 1c). Disruption of the open reading frame of ecp2 in three independent *S. musiva* MN-14 mutants resulted in notable physiological changes *in vitro*, including a slight increase in mycelial growth rate and a marked decrease in sporulation (Fig. 3a through c). When combined with the observed reductions in disease symptoms *in planta*, such as fewer cankers and reduced disease severity in specific *P. trichocarpa* genotypes (Fig. 4), these findings emphasize the importance of Ecp2 in key fungal processes, from growth and sporulation to host-pathogen interactions, collectively influencing disease development. The host specificity and role of *ecp2* in virulence was further confirmed in a GWAS analysis of the pathogen population on two different genotypes of *P. trichocarpa* (Fig. S1). It is also important to note that, regardless of the genotype that was inoculated, the paralog of *ecp2* is not associated with pathogen virulence (Fig. S2). Importantly, GWAS in this study was not used as a primary locus discovery tool, but rather as an independent line of evidence to prioritize candidate genes identified through transcriptomic profiling and functional validation. The convergence of differential expression, infiltration assays, gene disruption, and population-level association strongly supports SmEcp2 as a key virulence factor, while minimizing reliance on any single analytical approach. These findings align with broader observations on secreted fungal effector proteins, which are known to be key players in pathogenic interactions by enabling fungal growth and influencing other pathogenicity-related and physiological traits. For example, overexpression of the effector protein BAS1 in the rice blast fungus *M. oryzae* has been shown to significantly enhance fungal growth, spore formation along with virulence (41).

*Sphaerulina musiva* is considered a hemibiotrophic pathogen (27). Originally, hemibiotrophs were defined because of their initial haustorial feeding phase. However, the definition has now expanded to include pathogens with a long latent period. During latency, fungal hyphae first grow in the intercellular spaces between mesophyll cells and obtain nutrients from the host apoplast (42). Following this initial colonization, the pathogen switches to a necrotrophic phase, killing the host cells in the colonized tissue prior to reproduction (42). The symptom development observed on the stem, water-soaked lesions by 2 wpi, and necrosis at 3 wpi is consistent with this life history. We speculate that there are several potential roles for SmEcp2 in this interaction. For instance, if it is involved in the biotrophic phase of the *S. musiva* life cycle, then the protein would likely be recognized by a receptor in the host, leading to necrosis and resulting in resistance. However, we find no evidence of this as the SmEcp2-encoding gene was not expressed at early time points (28, 43). Alternatively, if this effector is involved in the necrotrophic phase of the *S. musiva* life cycle, the necrosis it induces likely results in susceptibility. The observed correlation between susceptibility and the protein’s ability to induce necrosis suggests that this effector plays a role in the necrotrophic phase of the hemibiotrophic lifestyle by inducing cell death (3, 42). Interestingly, SmEcp2 did not induce necrosis on all tested genotypes (Table 1). The most plausible explanation lies in the genetic diversity of the *P. trichocarpa* genotypes used in the infiltration assays, which were selected to represent the species’ range of diversity for association mapping studies (44). Therefore, SmEcp2 may act as an essential effector mediating virulence in certain genotypes but not in others, likely depending on genes found only in select host lines (Fig. S1).

As part of our investigation, we later examined the evolution and distribution of ECP2-like effector proteins across the fungal kingdom, uncovering critical insights into their diversity, ecological roles, and functional significance. In line with the work of Stergiopoulos et al. (31), we identified three main classes of ECP2 proteins, with only a few remaining unclassified. notably, Class 1 Ecp2 proteins were the most widespread across the fungal kingdom, often occurring in higher copy numbers (up to 21 copies) compared to Classes 2 and 3, which typically had only one or two copies per organism. Strains with five or more copies of Ecp2 Class 1 were predominantly found in the Sordariomycetes, 83% of which are identified as plant pathogens affecting hardwood trees such as *Hypoxylon* spp., *Biscogniauxia marginata*, and *Colletotrichum* spp. However, a small subset of non-pathogenic model fungi, including *Neurospora crassa* and *Podospora anserina*, also contained higher copy numbers of these genes. Our analysis further revealed three distinct subclasses of Class 3 ECP2 proteins, denoted as Classes 3.1, 3.2, and 3.3, each associated with unique ecological roles. For example, it is worth noting that 80% of the species containing the newly identified ECP2 Class 3.1 are opportunistic pathogens of humans, animals, plants, or insects. Similarly, most fungi (66%) containing ECP2 Class 3.2 are pathogens of animals, humans, or plants, or they live as parasites of other microbes. The other fungi within this class (22%) were either industrially important fungi for their ability to produce enzymes or used as biological control agents. The remaining fungi in this category (12%) are lesser known, and they are predicted to be soilborne, saprotrophic, or endophyte species found in association with plants. Finally, ECP2 Class 3.3 was predominantly found in biocontrol fungi (e.g., *Cordyceps militaris, Pochonia chlamydosporia,* and *Aspergillus fijiensis*), in fungal model organisms (e.g., *Podospora comata, Podospora anserina,* and *Neurospora crassa*) and in animal and vertebrate fungal pathogens (e.g., *Trichophyton equinum* and *Aspergillus turcosus*) (Fig. 5). These findings underscore the diverse roles of ECP2-like proteins across fungal taxa, linking their distribution to ecological and functional specialization.

Once thought to be unique to plant pathogens, effector proteins, including Ecp2, have since been identified in fungi with a variety of lifestyles, ranging from mutualists and endophytes to biocontrol agents (45–47). The increasing availability of fungal genomes has revealed that some effectors, such as Ecp2, are highly conserved across fungal taxa, suggesting roles that extend beyond host-pathogen interactions. This study demonstrates that while *S. musiva* utilizes Ecp2 to drive necrosis and disease in poplar trees, similar effectors are found across a wide range of fungal species, indicating their importance in both pathogenicity and broader ecological strategies. By uncovering the evolutionary patterns and functional diversity of Ecp2-like effectors, our findings provide a foundation for understanding how these proteins shape fungal ecology and host-pathogen interactions. These insights could inform the development of targeted disease management strategies, enhance crop resilience, and improve monitoring of fungal threats in response to emerging outbreaks and shifts in ecosystem dynamics.

## MATERIALS AND METHODS

### Pathogen culture

Isolations of *S. musiva* were made from individual branch cankers collected from a commercial nursery near Belle River in Minnesota (45.97 N, 95.19 W). Cankers were rinsed twice with sterile distilled water after soaking in a 5% NaClO solution for 2 min. The bark was removed from the stem, and a 5 mm piece of wood was excised from the margin between the healthy and necrotic tissue. The excised pieces were placed on Petri plates of KV-8 medium (180 mL V-8 vegetable juice, Campbell Soup Company, Camden, NJ, USA; 2 g calcium carbonate; 20 g agar; and 820 mL deionized water) (Stanosz and Stanosz 2002), amended with 100 mg L^–1^ of streptomycin (Streptomycin Sulfate USP, Amresco, OH, USA) and 240 mg L^–1^ chloramphenicol (Chloramphenicol USP, Amresco, OH, USA). Petri plates were grown at room temperature (20°C) under continuous light after being wrapped with Parafilm. Sporulating fungal colonies were transferred to new KV-8 Petri plates in 7 days. The species identity of fungal colonies appearing to be *S. musiva* was confirmed by morphology (48) and multilocus genotyping (49). Isolates were stored as mycelial plugs suspended in a 50% glycerol solution at −80°C in 2 mL cryotubes.

### Host propagation

Dormant cuttings of *P. trichocarpa* were collected from Oregon State University Research Farm near Corvallis, OR (44°35'18.4" N, 123°11'37.1" W). Dormant cuttings were cut into 10 cm lengths and planted in Cone-tainers (Ray Leach SC10 Super Cone-tainers, Stuewe and Sons, Inc., Tangent, OR, USA) measuring 3.8 cm in diameter and 21 cm deep, filled with growing medium (SunGro Professional Mix #8; SunGro Horticulture Ltd., Agawam, MA). The medium was amended with 38 g Osmocote slow-release fertilizer (15-9-12) (N-P-K) (7.0% NH_3_-N, 8.0% NO_3_-N, 9.0% P_2_O_5_, 12.0% K_2_O, 1.0% Mg, 2.3% S, 0.02% B, 0.05% Cu, 0.45% Fe, 0.23% chelated Fe, 0.06% Mn, 0.02% Mo, 0.05% Zn; Scotts Osmocote Plus; Scotts Company Ltd., Marysville, OH) after soaking in water for 48 h (50). Plants were grown in the greenhouse with an 18 h photoperiod supplemented with 600 W high-pressure sodium lamps and a temperature regime of 20°C/16°C (day/night). Plants were watered as needed and fertilized weekly with 20-20-20 (N-P-K) liquid fertilizer (Scotts Peters Professional; Scotts Company Ltd., Marysville, OH).

### Inoculations, mRNA isolation, and library preparation for RNA-seq

Before the RNA-seq experiment, six trees of *P. trichocarpa* (genotype GW 10998) were transplanted into 2 gallon pots filled with growing medium and 38 g Osmocote slow-release fertilizer. Experimental design included three biological replicates (three trees) at each time point: 2 wpi and 3 wpi. Three biological replicates (three cultures) were also included for the control samples of the pathogen grown in culture. The trees were point-inoculated with *S. musiva* isolate MN-14 by placing a 5 mm plug of sporulating mycelium over a lenticel, approximately 15 cm from the soil surface. The three biological replicates were collected at 2 and 3 wpi. Approximately 100 mg of symptomatic tissue from each inoculation point was harvested, placed in an MP Biomedicals Lysing Matrix tube, and flash frozen in liquid nitrogen. As a control, three fungal cultures of the same isolate were grown in liquid KV-8 media at room temperature on an orbital shaker and wrapped in aluminum foil. After 1 week of growth, the cultures were collected, flash frozen in liquid nitrogen, and placed in lysing matrix tubes. The cultures were used as an *in vitro* control. The frozen tubes were placed in a bead beater homogenizer, and the stem tissue was ground to a fine powder while still frozen.

The mRNA from each sample was extracted using the Dynabeads mRNA DIRECT Kit from Ambion. The manufacturer’s protocol was followed; however, to remove contaminants such as polyphenolics and polysaccharides that occur in high levels in *P. trichocarpa*, the Plant RNA Isolation Aid from Ambion, containing polyvinylpyrrolidone, was added to the lysing buffer. A chloroform cleanup step was also included before adding the supernatant to the Oligo Dynabeads. The concentration and quality of the mRNA were examined on an Agilent 2100 Bioanalyzer (Agilent Technologies, Santa Clara, CA). The three biological replicates from each time point were pooled together in equal concentrations, digested using RNase III, and the cDNA sequencing library was generated using the Ion Total RNA-Seq Kit v2 from Ion Torrent. Three separate chips, one for each time point, were run on the Ion Torrent Personal Genomics Machine (ThermoFisher, Waltham, MA), in the Department of Plant Pathology at North Dakota State University.

### Data analysis

The *S. musiva* genome (25, 27) and the *P. trichocarpa* genome (24) were used as references for mapping RNA-seq reads to their respective organisms. Expression level was estimated using the RPKM calculation in the CLC Genomics Workbench, using default parameters (Qiagen, USA). Read statistics were obtained with the command stats from seqkit v.2.8.2 (51). Quality of the reads was visualized using FastQC v.0.12.1 (52). Trimmomatic v.0.39 was used to trim the reads. Trimmed reads were mapped simultaneously to the genomes of *P. trichocarpa* and *S. musiva* SO2202 using STAR v.2.7.11b (53). Numbers of mapped reads were obtained with SAMtools (54). Comparisons were made between the 2 and 3 wpi treatments and the *in vitro* control. All genes that were upregulated in the *in planta* treatments relative to the control were considered. The small number of pathogen reads, at the early time points, limited the utility of a differential expression analysis for the identification of candidate genes. Thus, we focused on all genes that were upregulated in the inoculated tissue relative to the control. To identify a putative effector by protein expression via *P. pastoris* transformation and downstream validation, four criteria were used: (i) loci exhibiting differential expression concerning the control; (ii) blastx search (blast.ncbi.nlm.nih.gov) to examine sequence homology and predicted function; (iii) each sequence was examined for a predicted secretion signal cleavage site using the SignalP 4.1 Server algorithm (https://services.healthtech.dtu.dk/services/SignalP-5.0/); (iv) the presence of the transcript at both 2 wpi and 3 wpi; and (v) the translated protein is predicted to be <20 kDa in size. To test the feasibility of this approach, one gene was selected for transformation and subsequent protein expression.

### Transformation and protein expression

New cDNA was synthesized from undigested mRNA from 2 and 3 wpi samples using the GoScript Reverse Transcription System (First Strand cDNA synthesis). The cDNA was amplified by PCR using Q5 Hot Start High-Fidelity DNA Polymerase to amplify the candidate effector. Primers were Ecp2F1 (5′ ATAGAATTCATGCAGCTCAACAACGCCCTCCTC 3′) and Ecp2R1 (5′ ATAGGGCCCCAAGTCGGATCTCTGGACCCACCAG 3′). Reaction conditions were as follows: 1 min at 98°C, 35 cycles of 30 s at 98°C, 30 s at 66°C, 1 min at 72°C, and a final extension of 10 min at 72°C.

The PCR product was sent to GenScript USA Inc. to verify the sequence of the amplicon. Following confirmation, the PCR product was digested using ApaI and EcoRI (New England Biolabs Inc., Ipswich, MA), ligated into the pGAPZ A Expression Vector using T4 DNA Ligase (New England Biolabs Inc., Ipswich, MA), and transformed into DH5α competent cells. The cells were grown, and plasmid DNA was extracted using a QIAprep Spin Miniprep Kit. To confirm that the transformation was successful and that the candidate gene was “in-frame,” the construct was sequenced. Subsequently, the plasmid was linearized using AvrII (New England Biolabs Inc., Ipswich, MA) and then transformed into the *P. pastoris* expression vector using the *Pichia* EasyComp Transformation Kit (Thermo Fisher, Waltham, MA). PCR was used to confirm the successful transformation.

Protein expression was conducted by growing the transformant at 30°C for 72 h in a shaking incubator at 200 rpm. The expressed protein was purified by filtering the liquid culture through a Millipore Express PLUS Membrane Filter (0.45 µm) and processing through the HisPrep FF16/10 column (GE Healthcare, Fairfield, CT). Protein quality and quantity were assessed by visualization on a gel and using the NanoDrop ND-1000 UV-Vis Spectrophotometer.

### Infiltration assays

Two infiltration experiments were conducted using the purified protein. In the first experiment, dormant cuttings of *P. trichocarpa* trees were collected and planted at the North Dakota State University agricultural research greenhouse. The experimental design was a randomized complete block design with 7 genotypes and 4 blocks (7 genotypes × 4 blocks = 28 total trees). Trees were selected from a list of known resistant and susceptible genotypes used in a previous phenotyping experiment (BESC 184, BESC 234, GW 11024, HARA 26-2, HARC 26-5, HOMA 21-5, and SLMC 28-2). The second infiltration experiment was conducted at Oregon State University in an Enconair growth chamber with 16 h photoperiod using 60 W soft white incandescent bulbs and 4100 K fluorescent bulbs (Philips, Somerset, NJ). The temperature in the chamber was 21°C when infiltrations were conducted. The experimental design was a randomized complete block design with 6 genotypes and 4 blocks (6 genotypes × 4 blocks = 24 total trees). The genotypes used were BESC 184, BESC 22, BESC 234, BESC 801, GW 9805, and HARC 26-5. The purified protein (550 ng/µL) was infiltrated into the underside of each tree’s top-most fully expanded leaf. An empty vector and sterile distilled water control were used and infiltrated into the same leaves on the opposite side of the mid-vein. After 15 days, the leaves were removed and examined for necrosis.

### Disruption of the *ecp2* gene in *S. musiva* by CRISPR-Cas9-mediated HDR

#### gRNA and deletion cassette design

The protospacer sequence designed to target the *ecp2* gene (SEPMUDRAFT_146583) was the following: 5′-aaagattattgaccaagaag-3′, which corresponds to the fragment between nucleotide positions 215 and 235 on the Hce2 conserved domain. The location of the CRISPR RNA (crRNA) on the *ecp2* sequence is shown in Fig. 2a. This crRNA scored an MIT and CFD specificity scores of 100, a Doench efficiency score of 63, an Out-of-Frame score of 60, and an Off-Target score of 0. The crRNA and trans-activating crRNA (tracrRNA) (Cat. No. 1072533) molecules were purchased from IDT. To form the crRNA:tracrRNA duplex (gRNA), the Nuclease-Free Duplex Buffer (Cat. No. 1072570, IDT) was used according to the manufacturer’s instructions. The disruption cassette used as a template DNA for HDR consists of the vector ECP2-KO_S. musiva shown in Fig. 2b. The vector was synthesized through GenScript (https://www.genscript.com/) and comprises two 1,000 bp homology arms corresponding to the upstream and downstream flanking regions of the *ecp2* gene, along with the *ecp2* open reading frame disrupted at the designed gRNA cut site by the hygromycin resistance gene (*hph*) under the TrpC promoter and terminator from *Aspergillus nidulans*. Before the transformation, the vector was linearized using BsrGI.

#### PEG-mediated transformation and mutant screening

The gRNA (50 µM) and Cas9 nuclease (62 µM) complex (Cat. No. 1081059, IDT) and donor DNA (10 µg of linearized vector) were introduced together into the *S. musiva* strain MN-14 through PEG-mediated protoplast transformation according to Tannous et al. (26). Transformants were selected on SMM agar medium supplemented with hygromycin (100 µg/mL). As a negative control, the transformation procedure was also carried out on the protoplasts without adding any transforming agents (the template DNA and the components of the CRISPR-Cas9 system). Upon growth on selective media, the transformants were subsequently plated on new SMM-hygromycin plates and grown at 21°C for 10 days to obtain monoconidial isolates. Twenty transformed colonies were randomly selected and inoculated in GMM-yeast extract broth for genomic DNA extraction. Genomic DNA of the transformant strains was isolated as described (26). To confirm the disruption of the *ecp2* gene and insertion of the *hph* cassette at the targeted locus, the selected transformants were subjected to PCR using two primer pairs (ECP2-KO_conf5′F/R and ECP2-KO_conf3′F/R) that each have one primer in the flanking DNA and one primer in the antibiotic-resistance cassette. Primer sequences are presented in Table S3. To confirm the homokaryotic genotype of the purified Δ*ecp2* transformants, we performed PCR analyses targeting the ORF of the *ecp2* gene using the primer set ECP2-KO_ORF_F/R. This design yields a PCR product of 407 bp for the intact ORF and a product of 4,096 bp for the disrupted ORF. Three positive transformants identified by PCR were subjected to whole-genome sequencing using MinION Nanopore technology to confirm the absence of Cas9 off-target effects. To evaluate whether the CRISPR knockout of *ecp2* led to any off-target mutations, sequencing reads were mapped to the reference genome using the Geneious mapper implemented in Geneious Prime 2019 (https://www.geneious.com). SNPs were called from sites that had a read depth greater than 100× and visualized using TASSEL v.5 (https://tassel.bitbucket.io/).

#### Genomic library preparation and sequencing

Following the manufacturer’s instructions, the sequencing library was prepared using the NEBNext Companion Module for Oxford Nanopore Technologies Ligation Sequencing Kit (#E7180). Before library preparation, the gDNA was mechanically sheared to obtain 9 to 10 kb fragments using Covaris g-TUBEs (Covaris). Raw sequencing data files in fast5 format were transferred to an HP Z8 workstation equipped with an NVIDIA A5000 GPU. Basecalling was performed using the super-accurate basecalling model in Guppy v.6.2.1 (https://nanoporetech.com/software/other/guppy/history?version=6-2-1). Reads were filtered with Filtlong v.0.2.1 (https://github.com/rrwick/Filtlong) to remove sequences shorter than 2 kbp and the bottom 5% of reads by overall quality. These filtered reads were then assembled into contigs by Flye v.2.9 (55) and polished with Medaka v.1.6.0 (https://github.com/nanoporetech/medaka). Default settings were used for all software.

### Physiological analyses *in vitro* of *ecp2* disruption mutants

To evaluate the impact of mutations on colony morphology, growth, and conidiospore formation, *in vitro* physiological analyses were conducted on three distinct *ecp2*-disrupted mutant strains. The radial growth of the strains was assessed on potato dextrose agar (PDA) plates at 3, 7, and 10 days after the central inoculation of 10^6^ fresh conidia from the WT and mutant strains. Additionally, sporulation was evaluated on PDA agar plates using the agar overlay assay by Tannous et al. (56). Plugs were extracted on days 3, 7, and 10 post-inoculation, amended, homogenized in 1 mL of sterile water, and used for spore counts. The growth and sporulation experiments were tested with five technical replicates.

### Pathogenicity assays *in planta* of *ecp2* disruption mutants

The pathogenicity assay was conducted using the same 10 *P. trichocarpa* genotypes used in the infiltration assays (29). The trees were spray-inoculated with the three mutant isolates and one wild type (MN-14) *S. musiva* isolate (WT, Δ*ecp2-*1, Δ*ecp2-*5, and Δ*ecp2-*10) as detailed previously (29). For each treatment, 10 biological replicates of each genotype were used. The number of cankers and the disease severity score for each treatment were monitored and recorded 21 days post-inoculation. The disease severity score was measured using a scale from 1 to 5: 1 = no lesions; 2 = small necrotic lesions with swollen margins; 3 = small necrotic lesions; 4 = large necrotic lesions; 5 = stem girdled by lesions or sporulating lesions. This scale was adapted from LeBoldus et al. (57).

### Statistical analysis for fungal growth area, sporulation, and disease severity score

Statistical analyses were conducted using GraphPad Prism version 10.0.3 (275) for Windows 64-bit (GraphPad, San Diego, CA). Comparisons were made between treatments within each genotype, while differences across genotypes were not assessed. The normality of residuals for each genotype was evaluated using the Shapiro-Wilk test. Genotypes with a significant Shapiro-Wilk test result (*P*-value < 0.05) did not meet the normality assumption and were subsequently analyzed with a nonparametric Kruskal-Wallis test. For cases where the Kruskal-Wallis test was significant (*P*-value < 0.05), Dunn’s multiple comparison test was applied to compare treatments against the WT control. When the Shapiro-Wilk test was non-significant (*P*-value > 0.05), indicating that normality was met, a one-way ANOVA was conducted. If the one-way ANOVA was significant (*P*-value <0.05), Dunnett’s multiple comparison test was then used to compare all conditions against each other.

### Genome-wide association mapping for identification of genes associated with virulence

#### Obtaining and preprocessing the data

Illumina reads (100 bp, paired-end) were downloaded from the NCBI Sequence Read Archive for BioProject no. PRJNA543887, “Population genomics of *Sphaerulina musiva*” (58). These sequences represented 122 genotypes spread across the USA and Canada. In addition, the Sanger and Illumina reads used to generate the canonical reference assembly for *S. musiva* were downloaded from the DOE Joint Genome Institute’s Phytozome v12 (59), and the Illumina reads were retained for SNP calling. The reads were adapter-trimmed using bbduk using its default Illumina adapters.fa file (60). Reads were filtered using bbmap against a subset of the NCBI nonredundant nucleotide database to remove contaminants (61).

#### Mapping the reads to the reference

The trimmed and filtered Illumina reads for the 122 genotypes, plus the Illumina reference reads, were mapped to the *Sphaerulina musiva* reference using bwa-mem v.0.7.17 with the -M option (62). The resulting .bam files were sorted, and duplicates were marked and read groups labeled using samtools (63).

#### SNP calling

SNPs and small insertions/deletions were called using the GATK v.4.3 SNP calling pipeline (64). First, SNPs were called on a per-sample basis using the haplotype caller from GATK in haploid mode. Next, the gvcfs were merged using GATK’s CombineGVCFs. Then, SNPs/indels were called across the population (123 samples) using GATK’s joint SNP caller and gap-filtered using “bcftools filter -g 3 -G 10 -sFailGap.” This resulted in 9,447,185 SNPs, 1,701,729 insertions/deletions, with 1,991,918 multiallelic sites and 381,133 multiallelic SNP sites.

#### Estimating heritability and best linear unbiased predictors (BLUPs)

Four phenotypic traits were measured for 112 individuals, each belonging to one of five *Populus trichocarpa* host genetic backgrounds, with four replications per genotype in a greenhouse setting. Outliers were removed using the median absolute deviation (MAD) method, with data points having MAD >6 detected as outliers. Broad-sense heritability estimates ranged from 0.33 (for number of cankers on the poplar host genotype GW9824 background) to 0.66 (for height of the infected host genotype GW11026) (Table S4). BLUPs were estimated independently for each host genotype using mixed models with genotype and replication as random effects. Additionally, overall phenotypic BLUPs were estimated by including host genetic background as an additional random effect in the model. The lme4 package in R was used to fit the models and extract the BLUPs (65) for a total of 24 phenotypic BLUPs (4 traits × 5 individual host genetic backgrounds and 4 traits × 1 combined host genetic background), which were then used in the subsequent GWAS analyses. Phenotypic values were modeled as *y_ij_* = µ + *g_i_* + *r_j_* + ε*_ij_*, where µ is the overall mean, g_i_ is the random effect of the *i*th genotype, r_j_ is the random effect of the *j*th replicate, and ε*_ij_* is the residual error. Broad-sense heritability (*H*^2^) was estimated as $H^{2}=\frac{\sigma_{g}^{2}}{\sigma_{g}^{2}+\sigma_{\varepsilon }^{2}}$, where $\sigma_{g}^{2}$ is the genotypic variance and $\sigma_{\varepsilon }^{2}$ is the residual variance.

#### GWAS

As shown in Table S5, a total of 651,293 SNPs were retained for GWAS after excluding SNPs with a minor allele frequency less than 0.05, SNPs with more than 15% missing genotype calls, individuals with more than 15% missing SNP data, and SNPs exhibiting significant deviation from Hardy-Weinberg equilibrium (HWE *P*-value < 1 × 10⁻⁵⁰). A highly stringent HWE filter (*P* < 1 × 10⁻⁵⁰) was applied as a quality-control measure to remove only SNPs exhibiting extreme genotype distortions indicative of technical artifacts (e.g., mis-mapping or paralog collapse), while retaining variants with mild or moderate deviations from HWE. Genome-wide association analyses were conducted using one single-locus method (mixed linear model [MLM]) and three multilocus methods (fixed and random model circulating probability unification [FarmCPU], Bayesian-information and linkage-disequilibrium iteratively nested keyway [BLINK], and multiple loci mixed model [MLMM]), as implemented in the GAPIT3 software package in R (66). For each of the 24 phenotypic BLUPs, associations with genome-wide SNPs were tested using the following general MLM. The model included a genomic relationship matrix (GRM) as the variance-covariance structure for the random genetic effect, thereby accounting for background genetic relatedness among individuals (67):


(1) Y=Xβ+Zu+α+ϵ

where *Y* is the *Nx1* phenotypic vector with *N* being the sample size, β is the *Mx1* vector of additive SNP effects with *M* being the number of SNPs, ***X*** is the *N* × *M* incidence matrix, *u* is the *Nx1* vector of random genetic effects of genets, ***Z*** is the N × N genetic relationship matrix, α is the *Nx1* vector of random effects that include the GRM, and *ε* is the *Nx1* vector of residual random effects.

The three multilocus methods used either only the fixed effect models (BLINK) or the combination of fixed and random effects models (FarmCPU and MLMM).

BLINK employs an iterative process based solely on fixed effect models (68). In each iteration, the first fixed effect model (FEM) tests the association of each SNP with the trait, including as covariates all significant pseudo-quantitative trait nucleotides (QTNs)—SNPs previously identified as significant and not in linkage disequilibrium. The first FEM is represented as:


(2) $$y_{i}=S_{i1}b_{1}+S_{i2}\;b_{2}\;+\cdot \cdot \cdot \;+S_{ik}b_{k}\;+\;S_{ij}\;d_{j}\;+\;e_{j}$$

where *y_i_* is the phenotypic value of the *i*th individual; *S_i1_*, …, *S_ik_* are the genotypes of the *k* QTNs; *b1, …, bk* are the corresponding effects of the QTNs; *S_ij_* is the genotype of the *i*th individual and *j*th SNP; *d_j_* is the jth SNP effect; and *e_i_* is the random residual. The second FEM model (below) is the reduced version of equation 2 such that the *S_ik_b_k_* term, which tests for the association of the SNP with the phenotypic trait, is removed and is used to re-evaluate and update the set of pseudo-QTNs:


(3) $$y_{i}=S_{i1}b_{1}+S_{i2}\;b_{2}\;+\cdot \cdot \cdot \;+S_{ik}b_{k}\;+\;e_{j}$$

These steps are repeated until the set of pseudo-QTNs remains unchanged.

FarmCPU iteratively alternates between fixed effect and random effect models (69). In each iteration, the fixed effect model uses the current set of associated markers (pseudo-QTNs) as covariates to test SNP associations, while the random effect model uses a kinship matrix derived from these pseudo-QTNs to control for confounding due to relatedness and population structure. This iterative approach refines both the set of covariates and the kinship matrix.

MLMM extends the standard MLM by incorporating a GRM as the variance-covariance structure for the random genetic effect and by iteratively including significantly associated SNPs as fixed-effect covariates (66, 70). This procedure allows for stepwise model selection, with associated markers added at each step to account for the effects of multiple loci.

Quantile-quantile plots and genomic inflation factors (λ) were examined for all GWAS models and phenotypes to assess systematic inflation of test statistics. No evidence of widespread inflation was observed, indicating that population structure and relatedness were adequately controlled by the genomic relationship matrix and model framework.

#### SNP-to-gene assignment

We applied a Bonferroni-adjusted experiment-wise *P*-value threshold of 0.2 to identify GWAS signals for candidate prioritization rather than definitive locus discovery. This permissive threshold was selected intentionally to reduce false negatives in the context of a modest sample size, multiple GWAS models (MLM, FarmCPU, BLINK, and MLMM), and a strong prior hypothesis generated independently from transcriptomic and functional analyses. Under these conditions, the GWAS was used as a supporting line of evidence to corroborate candidates emerging from orthogonal data types rather than as a standalone discovery framework. Based on this criterion (raw *P*-value < 3.07 × 10⁻⁷, calculated as 0.2 divided by the number of tests, 651,293 SNPs), we detected 123 significant SNPs across four GWAS models and 19 phenotypic traits. Each significant SNP was assigned to its two nearest genes in the genome, typically the closest upstream and downstream genes, resulting in the identification of 237 unique genes (Table S6).

### Characterization of *ecp2* orthologs in Ascomycota

We used two comparative genomic approaches to explore the evolutionary origins of *ecp2* in *S. musiva*. First, we obtained all annotated publicly available sequences from NCBI on 20 April 2022. This data set consisted of 3,994 accessions representing 1,512 species. To avoid version differences in otherwise identical genomes and to manage taxonomic mislabeling (mostly caused by people entering isolate-level IDs as part of the species-level tag on NCBI), we selected a single representative from each species with the most ECP2 orthologs (detailed below) and removed all species that contained isolate-level identifiers (e.g., “Alternaria_sp_MG1”). We then scanned the protein files for each genome using hmmsearch with an e-value cutoff of 1 × 10^−4^ executed in HMMER v.3.3.2. All proteins containing a hit to the ECP2-specific PFAM domain, PF14856.7, were selected for further analysis. Resulting proteins were then classified using rules modified from Stergiopoulos et al. (31). Briefly, Class 1 and Class 2 ECP2 orthologs were defined by the presence of only the PF14856.7 domain, with the former requiring a length less than or equal to 400 AA and the latter including lengths between 401 and 1,300 AA. Class 3 orthologs were at least 1,000 AA in length and contained the PF14856.7 domain in addition to the PF01476.21 LysM peptidoglycan-binding domain, PF00704.29 GH18 chitinase domain, and the PF00187.20 chitin-binding domain. Preliminary analysis of resulting classifications demonstrated that some proteins contained only a subset of the Class 3 domains and were thus defined as follows: Class 3.1 contained PF00187.20, PF00704.29, PF14856.7; Class 3.2 contained PF00704.29, PF01476.21, PF14856.7; Class 3.3 contained PF00704.29, PF14856.7. A small number of proteins did not fit into any of these classifications, but no individual group had large representation (i.e., the additional domains present were very diverse). These unclassified proteins were put into a catch-all classification of “Class?.” The presence of a putative ortholog was mapped to a quartet-based phylogeny representing a single individual for every species, as modified from Nickles et al. (30).

Second, we downloaded a data set of annotated fungal proteomes in the Pezizomycotina from RefSeq using the GenBank download data set tool (355 individual genomes; Table S7) and used OrthoFinder v.1.0.6 with a Diamond search and the default inflation parameter to group orthologs and paralogs of gene families in the Ascomycota. We identified orthogroups homologous to *ecp2* based on two genes in *S. musiva* (SEPMUDRAFT_146583 and SEPMUDRAFT_149875). We aligned single-copy orthologs identified by OrthoFinder with MAFFT v.7.508, concatenated these genes with FASconCAT-G, and searched for a maximum likelihood tree with IQTree v.2 (71) and a test for the best model. Gene copy number was counted for each examined taxon and plotted in a heatmap over the final phylogeny using ETE3 in Python (72).

## Data Availability

The authors affirm that all data necessary for confirming the conclusions of the article are present within the article, figures, tables, and repository. Raw sequencing reads used in this study were deposited at the NCBI Sequence Read Archive (SRA): SRR37042235 (2wpi), SRR37042237 (3wpi), and SRR37042236 (control).

## SUPPLEMENTAL MATERIAL

Supplemental figures mbio.03120-25-s0001.docx Fig. S1 and S2.

Data set S1 mbio.03120-25-s0002.xlsx Upregulated genes.

Table S1 mbio.03120-25-s0003.xlsx Summary statistics of IonTorrent RNA-seq reads.

Table S2 mbio.03120-25-s0004.xlsx Summary of whole-genome sequencing read metrics.

Table S3 mbio.03120-25-s0005.xlsx Primers used to confirm the insertional mutation of *ecp2* in *S. musiva* strain MN-14.

Table S4 mbio.03120-25-s0006.xlsx Broad-sense heritability estimates for *Sphaerulina musiva* phenotypic traits with *Populus trichocarpa* as the host genetic background.

Table S5 mbio.03120-25-s0007.xlsx Quality control applied to SNP variants in *Sphaerulina musiva*.

Table S6 mbio.03120-25-s0008.xlsx Significant associations.

Table S7 mbio.03120-25-s0009.xlsx ECP2 copy number in analyzed genomes.

ASM does not own the copyrights to Supplemental Material that may be linked to, or accessed through, an article. The authors have granted ASM a non-exclusive, world-wide license to publish the Supplemental Material files. Please contact the corresponding author directly for reuse.
