# Supplementary material for: Transcriptomic and functional analyses uncover a conserved effector driving genotype-dependent virulence in the Sphaerulina musiva-Populus trichocarpa interaction
Source: mBio. 2026 Apr 16;17(5):e03120-25. doi: 10.1128/mbio.03120-25 (PMC13170265; doi:10.1128/mbio.03120-25)
Supplement: Supplemental figures — Fig. S1 and S2. [file mbio.03120-25-s0001.docx]

**Supplemental figures:**


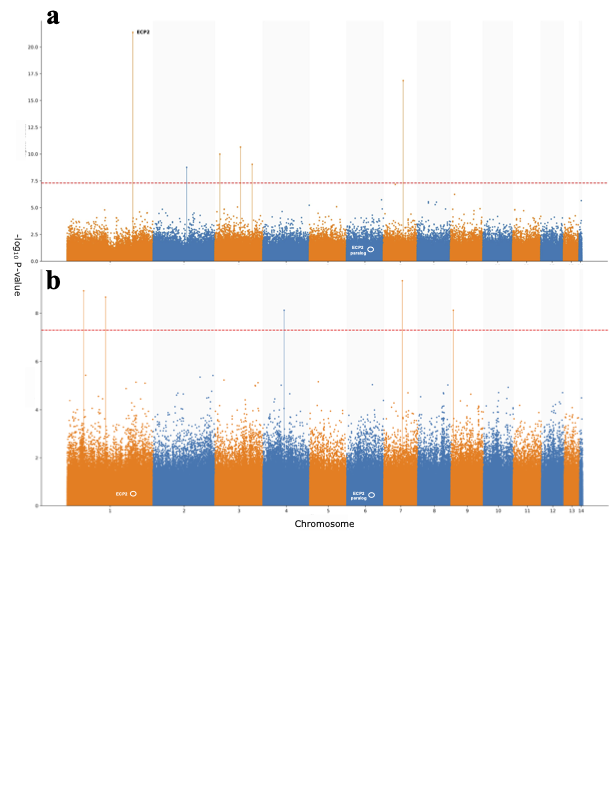


**Fig. S1:** Manhattan plots of the *S. musiva* genome identifying host specific virulence associated loci for two *Populus trichocarpa* genotypes (A) GW9823 and (B) CMBF28.1 inoculated with 112 isolates of *S. musiva*. (A) The significant association of *ecp2* (SEPMUDRAFT_146583) with the virulence of *S. musiva* on GW9823. (B) The absence of a significant association with *ecp2* (SEPMUDRAFT_146583) and *S. musiva* virulence on CMBF28.1. The position of the *ecp2* paralogue (SEPMUDRAFT_149875) on both (A) GW9823 and (B) CMBF28.1 is circled in white. Each dot on the Manhattan plots corresponds to a marker, its level of significance, and its physical position. The red lines represent the Bonferroni corrected threshold for a significant association corrected for 651,293 comparisons.


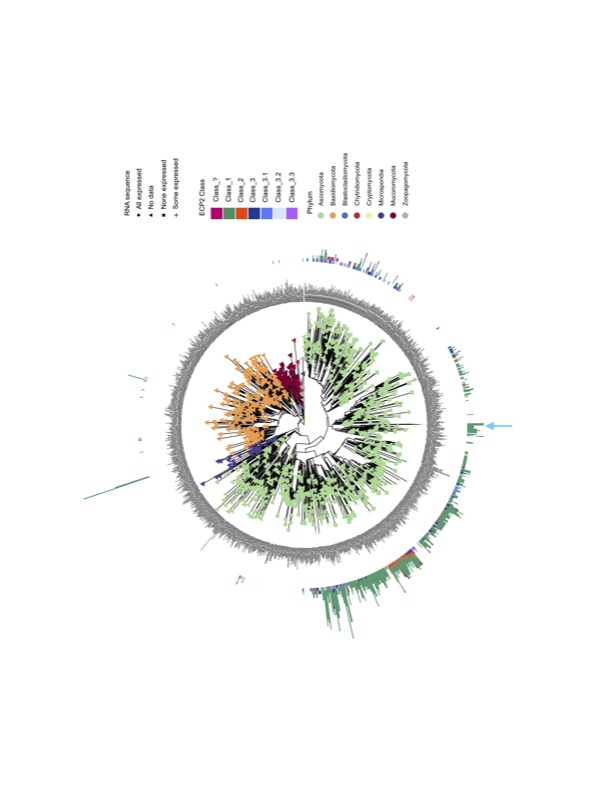


**Fig. S2:** The phylogenetic relationship of all publicly available annotated fungal genomes that were scanned for ECP2 orthologs are depicted in a quartet-based phylogeny as modified from previous work ^66^. The tree has been subset to contain only a single representative of each species, this representative was selected based on the highest ECP2 ortholog count (or randomly selected when multiple isolates of the same species had the same count). Note that while all Phyla are monophyletic and most are correctly placed in the phylogeny, the placement of Microsporidia is erroneous compared to previous work; this issue has no impact on our interpretation. Counts of different ECP2 Class variants (see methods) are depicted in a stacked bar graph around the outer rim of the phylogeny. The phylogeny is rooted at the only representative of the early-divergent lineage of Cryptomycota, *Rozella allomycis*, a species whose genome contains a single Class 1 ECP2 ortholog. In cases where RNA seq were available for a species displayed in the tree, we compared the raw gene counts of ECP2 orthologs between genomes and transcriptomes to determine if any, some, or all genes may be expressed. Analysis of RNA-seq data demonstrates that expression of ECP2 orthologs is widespread across the phylogeny. However, because of technical constraints, this analysis was performed at the species level and does not reflect a direct pairing of specific orthologs; we found no instances where there were more ECP2 orthologs in expression data than in genomic sequences. The position of *S. musiva* in the tree is indicated with a blue arrow.
